# Validation of a novel questionnaire for assessing occupational exposure to organophosphate pesticides in Chilean agricultural workers

**DOI:** 10.3389/ftox.2025.1588408

**Published:** 2025-08-18

**Authors:** Liliana Zúñiga-Venegas, Natalia Landeros, Floria Pancetti, Sandra Cortés, Boris Lucero, Ana M. Brito, Ian S. Acuña-Rodríguez, María Teresa Muñoz-Quezada

**Affiliations:** ^1^ Centro de Investigación y de Estudios Avanzados del Maule (CIEAM), Universidad Católica del Maule, Talca, Chile; ^2^ Laboratorio de Investigaciones Biomédicas (LIB), Facultad de Medicina, Universidad Católica del Maule, Talca, Chile; ^3^ Centro Oncológico, Facultad de Medicina, Universidad Católica del Maule, Talca, Chile; ^4^ Laboratorio de Neurotoxicología Ambiental, Universidad Católica del Norte, Coquimbo, Chile; ^5^ Escuela de Salud Pública UC. Facultad de Medicina. Centro Avanzado de Enfermedades Crónicas (ACCDIS), Pontificia Universidad Católica de Chile, Santiago, Chile; ^6^ Centro de Desarrollo Urbano Sustentable (CEDEUS), Pontificia Universidad Católica de Chile, Santiago, Chile; ^7^ Centro de Investigación en Neuropsicología y Neurociencias Cognitivas (CINPSI - Neurocog), Universidad Católica del Maule, Talca, Chile; ^8^ Departamento de Salud del Personal, Hospital Regional de Talca, Talca, Chile; ^9^ Centro de Ecología Integrativa, Vicerrectoría Académica, Dirección de Investigación, Universidad de Talca, Talca, Chile; ^10^ Programa de Epidemiología, Escuela de Salud Pública, Facultad de Medicina, Universidad de Chile, Santiago, Chile

**Keywords:** organophosphate pesticides, occupational exposure, cholinesterase inhibition, DAP metabolites, questionnaire validation, Chile

## Abstract

**Background:**

Unintentional pesticide poisoning is a global health concern, disproportionately affecting agricultural workers in developing countries due to inadequate regulations and limited access to protective equipment. While questionnaires offer a cost-effective alternative for assessing organophosphate (OP) pesticide exposure compared to urinary (e.g., Dialkyl Phosphates, DAPs) or blood biomarkers (e.g., acetylcholinesterase, AChE, and butyrylcholinesterase, BChE), these tools require validation against gold-standard methods. This study validated a questionnaire assessing occupational OP exposure among Chilean agricultural workers in the Maule region, contrasting its performance against DAP levels and AChE and BChE inhibition.

**Methods:**

A longitudinal study was conducted with 51 agricultural workers. Urinary DAPs, measured via liquid chromatography–tandem mass spectrometry, AChE, and BChE activities, determined by Ellman’s method, were measured before (T0) and during (T1) the spray season. The questionnaire was administered at T1. Sensitivity, specificity, predictive values, and Receiver Operating Characteristic (ROC) curve analyses were performed to assess the accuracy.

**Results:**

Urinary DAP levels and AChE inhibition increased in T1 (from 6.54 ± 4.66 to 12.39 ± 9.88 μg/g creatinine, *p* = 0.004, and from 2.26E-3±6.53E-4 to 1.44E-3±2.73E-4 mmol/min^-1^*mgProt^-1^, *p* < 0.001, respectively), with AChE inhibition (30.99%) exceeding Chilean regulatory threshold. The questionnaire score correlated with AChE inhibition (*p* = 0.0063) but not with BChE inhibition or DAP levels. Sensitivity was 64%, and specificity improved from 56% to 71% when using a 20% AChE inhibition threshold instead of a 30%.

**Conclusion:**

Agricultural workers in the Maule region are exposed to OP pesticides. The questionnaire shows potential as a screening tool for occupational exposure. These findings highlight the need to reassess the Chilean regulatory limits and refine the tool to enhance risk assessment and intervention planning.

## 1 Introduction

Occupational pesticide poisoning remains a major public health concern, particularly in agricultural settings where workers are disproportionately exposed to toxic chemicals (Panis et al., 2022). It is estimated that 25 million agricultural workers worldwide experience unintentional pesticide poisoning annually, with low- and middle-income countries being the most affected due to limited regulations and insufficient access to personal protective equipment (PPE) (Alavanja, 2009). The World Health Organization (WHO - World Health Organization, 2019) underscores the risks of acute pesticide toxicity, particularly in regions with intensive pesticide use and inadequate safety measures.

Among these toxic chemicals, organophosphate (OP) pesticides are widely used in agriculture due to their effectiveness in pest control. However, OP pesticide exposure poses significant health risks to farm workers because of their acute and chronic toxicity (Shekhar et al., 2024).

Occupational exposure to OPs has been associated with a variety of health issues, including neurotoxic effects (e.g., cognitive impairment and neurodegenerative disorders), which arise primarily through the inhibition of acetylcholinesterase (AChE) and butyrylcholinesterase (BChE), key enzymes for the breakdown of acetylcholine, a critical neurotransmitter for nerve function (Aroniadou-Anderjaska et al., 2023). The resulting accumulation of acetylcholine leads to a cascade of neurological and physiological disruptions, ranging from mild symptoms such as headaches and nausea to severe effects, including paralysis and respiratory failure (Lott and Jones, 2022).

AChE inhibition is a well-established biomarker for OP poisoning due to its sensitivity, long half-life, and direct association with neurotoxicity, making it particularly useful for assessing both acute and chronic exposure (Strelitz et al., 2014). In comparison, BChE has a faster turnover rate, reflecting more immediate, acute exposures (Chambers and Chambers, 1990). Consequently, monitoring AChE and BChE activity remains critical for evaluating OP pesticide exposure and its health effects (Johnstone et al., 2007; Kapka-Skrzypczak et al., 2011; Strelitz et al., 2014; Ramírez-Santana et al., 2020a).

Agricultural workers are particularly vulnerable due to their direct and frequent contact with these chemicals, exacerbated by insufficient safety measures, limited training, and restricted access to PPE (Panis et al., 2022; Zúñiga-Venegas et al., 2022). Despite the well-documented risks, cost-effective and accessible exposure assessment tools remain scarce, particularly in low-resource settings.

Accurate exposure assessment is essential for effective intervention, yet current methods present challenges. Common biomonitoring approaches for OP pesticide exposure include urinary Dialkyl Phosphate metabolites (DAPs) and cholinesterase (AChE/BChE) inhibition measurements, which provide reliable data but are often costly, invasive, and difficult to implement on a large scale (Coble et al., 2005).

Given these limitations, structured questionnaires offer a practical and affordable alternative for assessing pesticide exposure by collecting information on pesticide use, work habits, and PPE practices. However, their accuracy depends on proper validation to ensure reliable results (Ohlander et al., 2020). Combining questionnaire data with occupational and environmental factors through exposure algorithms can improve precision (Oltramare et al., 2023). Studies comparing questionnaires with biomonitoring, using AChE and BChE inhibition as gold standard, have shown varying results, highlighting the need for tools that capture both acute and chronic exposure (Filippi et al., 2021; Sontakke and Kalantri, 2023; Chaudhary et al., 2019).

Nowadays, Chile is positioned as a key food producer in the southern hemisphere (FAO–Food and Agriculture Organization–Chile, 2024), and the Maule region, characterized by its Mediterranean clime, represents the second-largest agricultural area in the country, after Araucanía region, with extensive OP pesticides usage (SAG - Servicio Agrícola y Ganadero, 2023). The Maule region accounted for 25% of reported acute pesticide poisoning cases nationwide in 2023 (MINSAL - Ministerio de Salud. Gobierno de Chile, 2023), making it an ideal setting to assess the effectiveness of exposure assessment tools in a real exposure scenario. In this region, effective assessment of agricultural workers’ exposure is essential for implementing targeted prevention and protection strategies.

Prior exposure questionnaires, such as those used in Argentina and South Asia (Filippi et al., 2021; Chaudhary et al., 2019; Sontakke and Kalantri, 2023), often lacked validation with biomarkers, and frequently failed to integrate home exposures, workplace hygiene, PPE practices, and chronic exposure conditions, especially in small-scale agricultural context. In contrast, the QOP-UCM questionnaire (Muñoz-Quezada et al., 2019a) was designed to overcome these gaps by incorporating these multidimensional aspects.

In this context, this study’s novel contribution lies in the comprehensive validation of the QOP-UCM questionnaire, designed to measure occupational exposure to OP pesticides in the Maule region, employing validated biomarkers as the gold standard (AChE and BChE inhibition) to assess the questionnaire’s performance. These findings provide crucial information for improving occupational health programs and policies aimed at protecting farm workers in this high-risk region.

## 2 Materials and methods

### 2.1 Study design, population and sample size

A longitudinal study was conducted among agricultural workers, consisting of two monitoring sessions. The first session took place before the fumigation period, during June and July (autumn-winter) (T0), and the second was conducted during the OP pesticide application period in December and January (summer) (T1). Based on the DAPs levels reported by Ueyama et al. (2012) in a longitudinal study of apple farmers, which showed high detection rates (>98%) and significant season differences, a sample was calculated assuming 95% confidence, 5% error, and a 20% dropout rate. The resulting estimate suggested that fewer than 30 participants would be sufficient but could be inadequate for achieving robust statistical power. To increase robustness and account for potential losses, we enrolled 57 agricultural workers (*n* = 57), the maximum feasible within budgetary and logistical constraints.

**FIGURE 1:**
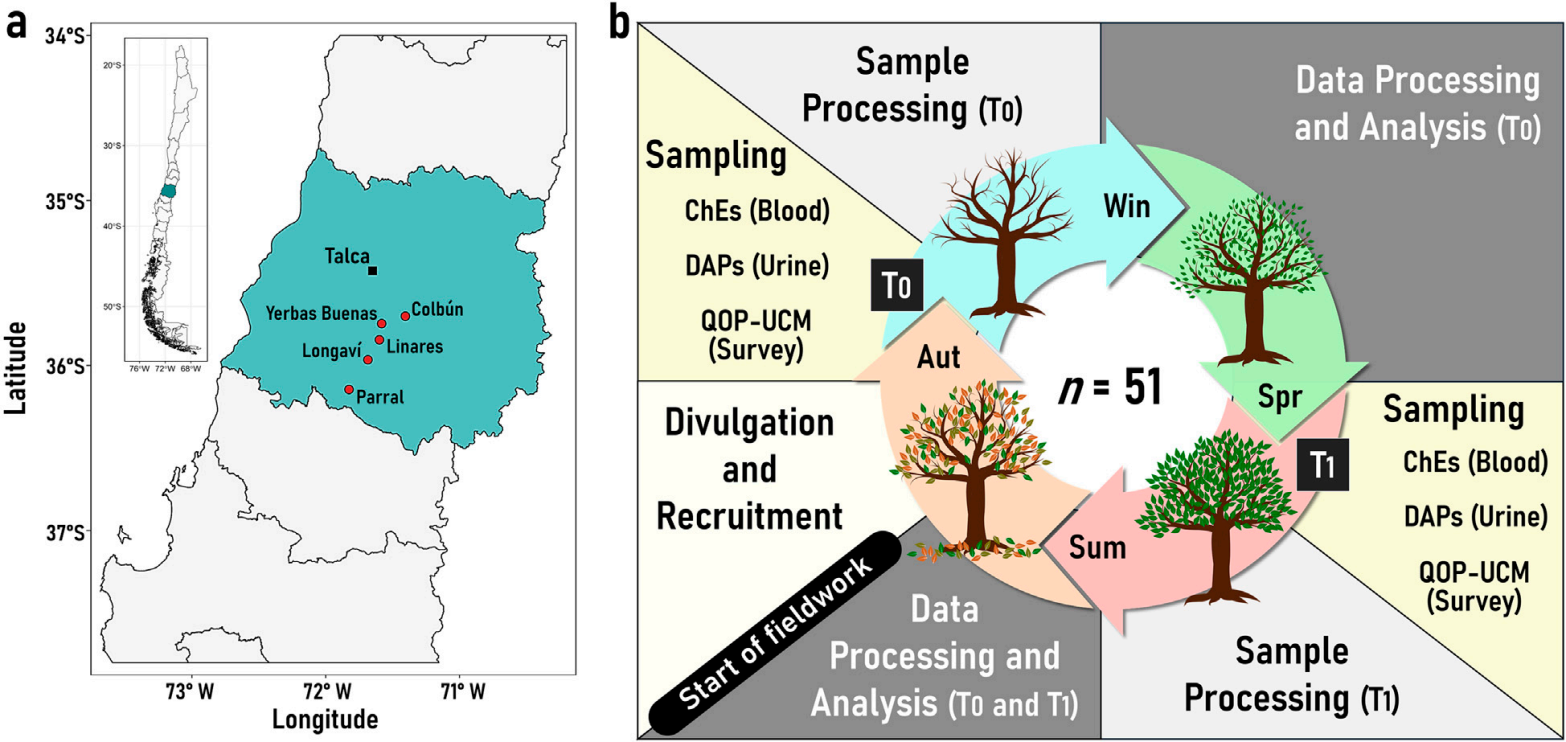
Map and study design diagram. Panel **(a)** shows the Maule region of Chile, with its capital city, Talca, highlighting locations of farmworkers recruitment: Yerbas Buenas (*n* = 7), Longaví (*n* = 10), Parral (*n* = 16), Linares (*n* = 3), and Colbún (*n* = 21), represented by red points. Panel **(b)** illustrates the annual study process with two monitoring sessions: T0 or before spray season (June–July, autumn–winter), and T1 or during spray season (December–January, summer). The process included the following phases: divulgation and recruitment, sampling in T0, sample processing, data analysis, sampling in T1 processing and analysis. Abbreviations: QOP-UCM, Questionnaire of occupational exposure to organophosphate pesticides; ChEs, Cholinesterase activity measurement in blood; DAPs, Dialkyl Phosphates metabolites in urine; Win, Winter; Aut, Autumn; Sum, Summer; Spr, Spring. This figure includes a public domain image under a Creative Commons Zero (CC0) license (www.needpix.com).

Figure 1 illustrates the distribution of sampling locations where participants were recruited. The rural areas of Parral (*n* = 16), Linares (*n* = 3), Longaví (*n* = 10), Colbún (*n* = 21), and Yerbas Buenas (*n* = 7), were selected due to their intense agricultural activity in the Maule region in Chile, where the primary crops include fruits, vegetables, and vineyards (Figure 1).

Workers were selected based on their occupational exposure to pesticides and were contacted through the National Institute of Agricultural Development (INDAP), under the Ministry of Agricultural of Chile. Recruitment was carried out via the Local Development Program (PRODESAL), which maintains records of small-scale farmers who utilize government-subsidized agrochemicals.

Men and women between 18 and 65 years of age were invited to voluntarily participate. Eligible participants were seasonal workers who work in direct contact with pesticides, specifically those involved in applying, preparing, formulating, and/or mixing OP pesticides intensively during the summer season while having no direct OP exposure during winter. Workers diagnosed with chronic diseases were excluded from the study. All participants signed an informed consent form, approved by the Scientific Ethics Committee of the Universidad Católica del Maule (#19/2022).

### 2.2 Fieldwork and sampling

All participants were interviewed for both questionnaire administration and biological sample collection before (T0) and during (T1) the spray season. Questionnaires were administered either at the workplace or at the worker’s homes and gathered data on sociodemographic characteristics, lifestyle, medical history, pesticide use and occupational exposure.

For sample collection, 4 mL of blood were obtained drawn via venous puncture, and 20 mL of first-morning urine were collected 2 days after blood sampling to account for metabolites clearance. Samples were transported at 4°C to the Biomedical Science Laboratory at the Universidad Católica del Maule. Blood samples were processed within 24 h to separate blood cells from plasma via centrifugation. Each fraction was stored in three aliquots at −20°C before being shipped on dry ice to the Environmental Neurotoxicity Laboratory at Universidad Católica del Norte (Coquimbo, Chile) for cholinesterase activity assessment. Urine samples were also divided into three aliquots and stored at −20°C. Two aliquots were subsequently shipped on dry ice to the U.S. Centers for Disease Control and Prevention (Atlanta, Georgia, United States) for DAP metabolite measurement.

### 2.3 Assessment of occupational exposure to OPs

#### 2.3.1 Dialkyl phosphate metabolites (DAPs) measurement

Following previously validated techniques (Jayatilaka and Restrepo, 2019), concentrations of OPs pesticides, including diethyl phosphate - DEP; diethyl thiophosphate - DETP; diethyl dithiophosphate - DEDTP; dimethyl phosphate - DM; dimethyl thiophosphate–DMTP, were quantified in urine. Briefly, frozen samples were thawed and enzymatically deconjugated with β-glucuronidase/sulfatase in sodium acetate buffer at 37°C for 17 h. The analytes were extracted by solid-phase extraction (SPE) using Strata-X-AW cartridges and eluted with 2% ammonium hydroxide in methanol. After drying under nitrogen, samples were reconstituted in 95:5 water:methanol-acetonitrile for analysis.

Metabolic concentrations were measured using high-performance liquid chromatography (HPLC) on an Agilent 1,290 system coupled with tandem mass spectrometry (AB Sciex 5,500 Qtrap system) equipped with TurboIonSptay^®^ source in negative ion mode (Applied Biosystems). The method employed scheduled multiple reaction monitoring (sMRM) and isotope dilution for quantification. The limits of detection (LOD) for each metabolite were 0.1 μg/L. Urinary creatinine concentrations were measured using the Jaffé-reaction, applying kinetic colorimetric techniques (Creatinine liquocolor test, Human Diagnostic Worldwide) to adjust OP metabolite levels (μg metabolite/g creatinine), accounting for hydration status variability among participants. DAP concentrations below the LOD were assigned the value of LOD/√2, as per (Hornung and Reed, 1990).

#### 2.3.2 Cholinesterase´s activity (AChE and BChE) measurement

Biomarker activities were measured according to Ramírez-Santana et al. (2015), Ramírez-Santana et al. (2018). Briefly, the blood sample was centrifuged to separate plasma and erythrocyte fractions and then was frozen at −80°C until the measurement. One aliquot of blood cells and one of plasma per volunteer were thawed on ice and combined with a Tris-DTT buffer (100 mM). Mechanical lysis was performed using a tuberculin syringe. The resulting lysate (erythrocyte membranes) was centrifuged at 10,000 rpm for 30′ at 4°C, washed twice with 1x PBS, and resuspended in 1x PBS buffer with 0.5% Triton X-100. AChE activity was measured using the Ellman´s colorimetric method using acetylthiocholine iodide as the substrate and DTNB reagent (Ellman et al., 1961), with absorbance read at 412 nm. Results were expressed as specific activity normalized by the erythrocyte membrane protein concentration (mmol x min^-1^ x mg protein^−1^). BChE activity was measured from undiluted plasma using a similar approach, with specific activity also expressed in mmol × min^-1^ × mg protein^−1^. Reaction conditions were standardized to 30°C with readings taken at 406 nm every 15 s for 2 min, using acetylthiocholine or butyryl thiocholine as substrates and 0.25 mM DTNB Protein concentrations in plasma and erythrocytes membranes were quantified using the bicinchoninic acid (BCA) method (Smith et al., 1985).

#### 2.3.3 QOP-UCM questionnaire for OP exposure assessment

Occupational exposure was also estimated using the QOP-UCM questionnaire, developed by researchers at the Universidad Católica del Maule (Muñoz-Quezada et al., 2019a). The complete version of the questionnaire is available in Supplementary Table S1. The questionnaire consisted of 37 items designed to assess occupational OP exposure among agricultural workers, based on previous surveys from the Institute of Public Health of Chile (ISPCH - Instituto de Salud Pública de Chile, 2014), enriched with additional questions addressing both acute and chronic exposure scenarios.

This instrument has demonstrated strong internal consistency (Cronbach’s alpha = 0.95) and factorial validity. Four dimensions were identified through exploratory factor analysis with Varimax rotation, explaining 68% of the variance: (1) labor conditions during application of OPs (e.g., “How many years have you applied pesticides?“), (2) use of personal protective equipment (e.g., “Do you use protective gloves when handling pesticides?“), (3) workplace conditions related to OP exposure (e.g., “Does your workplace have a washbasin available?“), and (4) home conditions that facilitate exposure (e.g., “Do you use OPs at home?“). Questionnaire responses were scored using a scale from 0 to 2 or 3 depending on the item, with higher scores indicating greater risk of exposure. The first three factors demonstrated reliability above 75%, while the fourth reached 60%.

The total questionnaire score is 54 points, with higher scores indicating greater OP exposure. During the questionnaire validation, farmworkers achieved a median score of 32 points, with an interquartile range (IQR) of 25% of 25.3 points. This median was used as a cutoff point in subsequent analyses.

### 2.4 Statistical analysis

Exploratory (descriptive) data analysis was performed to compare T0 and T1 responses, and inferential analyses were conducted to evaluate variables predicting cholinesterase inhibition and the questionnaire-based OP exposure estimates.

Comparisons between T0 and T1 included 51 individuals, as six workers were lost to follow-up. Boxplots were created to visualize the total level of Diethyl Alkyl Phosphates (ΣDAPs) metabolites and cholinesterase inhibition by season. Seasonal comparisons (T0 vs. T1) were performed using the Wilcoxon signed rank test.

For AChE and BChE activity changes one-sample t-test (test value = 0) were conducted. Additional comparisons of cholinesterase inhibitions, change in ΣDAPs concentration, and QOP-UCM scores were analyzed using the Student’s t-test.

Bivariate analyses were used to establish associations between exposure levels according to cholinesterase inhibition and QOP-UCM score. Multivariate regression models, adjusted for age, sex, years of residence, and PPE use, were built using the “enter” method for variable selection (all variables in a single step) to establish the associations. Variables included in the multivariate regression models were selected based on their theoretical relevance and statistical significance in bivariate analyses. Multicollinearity was evaluated using variance inflation factors (VIF), with all values below 2, indicating acceptable independence among predictors.

Finally, sensitivity, specificity, predictive values and ROC curve analyses were performed, contrasting the AChE inhibition thresholds of 20% and 30%. Statistical analyses were conducted using R (v4.3.0) (R-Core Team, 2023) and IBM SPSS Statistic 25 (SPSS Inc. Chicago, IL).

## 3 Results

### 3.1 Study population profile

Of the 57 agricultural workers initially recruited, six (two who declined and four lost to follow-up) were excluded, resulting in a final sample to 51. The study group had a mean age of 47.8 years, with an average of 10.2 years of education. Most workers (70.2%) were full-time farmers, while 12.3% worked seasonally. Participants had an average of 24 years of experience handling pesticides, with the majority involved in mixing (87.7%), applying pesticides (93%), and machine washing (84%). The primary crops cultivated by participants were raspberries (59.9%), blueberries (31.6%), and blackberries (21.1%) (Table 1).

**TABLE 1:** Sociodemographic, task, and health characteristics of study group (*n* = 57).

Characteristics of agricultural workers group		Mean ± SD or frequency (%)
Age (years, x̅ ± SD)		47.8 ± 10.3
Sex (*n*, %)	Male	44 (77.2)
	Female	13 (22.8)
Study level (years of scholarity, x̅ ± SD)		10.2 ± 2.5
Time living in rural area (years, x̅ ± SD)		38.4 ± 15.5
Time working in contact with pesticides (years, x̅ ± SD)		24.0 ± 13.5
Type of pesticide usually used (*n*, %)	Fungicide	20 (35.1)
	Insecticide	33 (57.9)
	Herbicide	34 (59.6)
	Others	6 (10.5)
Current job (*n*, %)	Farmer	40 (70.2)
	Seasonal farmworker	7 (12.3)
	Tractor driver	7 (12.3)
	Other agricultural services	3 (5.3)
Working task (*n*, %)	Pesticide handler (mixer)	50 (87.7)
	Pesticide applicator	53 (90.3)
	Machine washers	48 (84)
	Other tasks	7 (12.3)
Crop types (*n*, %)	Raspberries	29 (50.9)
	Blueberries	18 (31.6)
	Blackberries	12 (21.1)
	Corn/wheat	10 (17.5)
	Tomato	8 (14.0)
	Others	29 (28.1)
Use of personal protection elements (*n*, %)	Simple mask	15 (26.3)
	Filter mask	33 (57.9)
	Goggles	33 (57.9)
	Gloves	39 (68.4)
	Boots	34 (59.6)
	Coverall	26 (45.6)
	Others	16 (28.1)
Acute intoxication by pesticides (*n*, %)^a^		15 (26.3)
Alcohol consumption (*n*, %)	No	10 (17.5)
	Yes	47 (82.5)
Smoking habit (*n*, %)	No	46 (80.7)
	Yes	11 (19.3)

^a^ Acute intoxication refers to self-reported incidents where participants experienced pesticide-related symptoms (e.g., dizziness, nausea, vomiting, headache) severe enough to warrant medical attention or work interruption.

Regarding PPE use, 68.4% of workers reported using gloves, 59.6% used boots, and 57.9% wore a mask with a filter. However, a significant proportion (15%) of mask users either never changed their filter or were unaware of how to do so. In general, 21% of workers either used only one type of PPE or did not use PPE at all. Additionally, 26.3% of workers reported experiencing acute pesticide poisoning symptoms at least once during their working history (Table 1).

### 3.2 Biomarkers’ results

**FIGURE 2:**
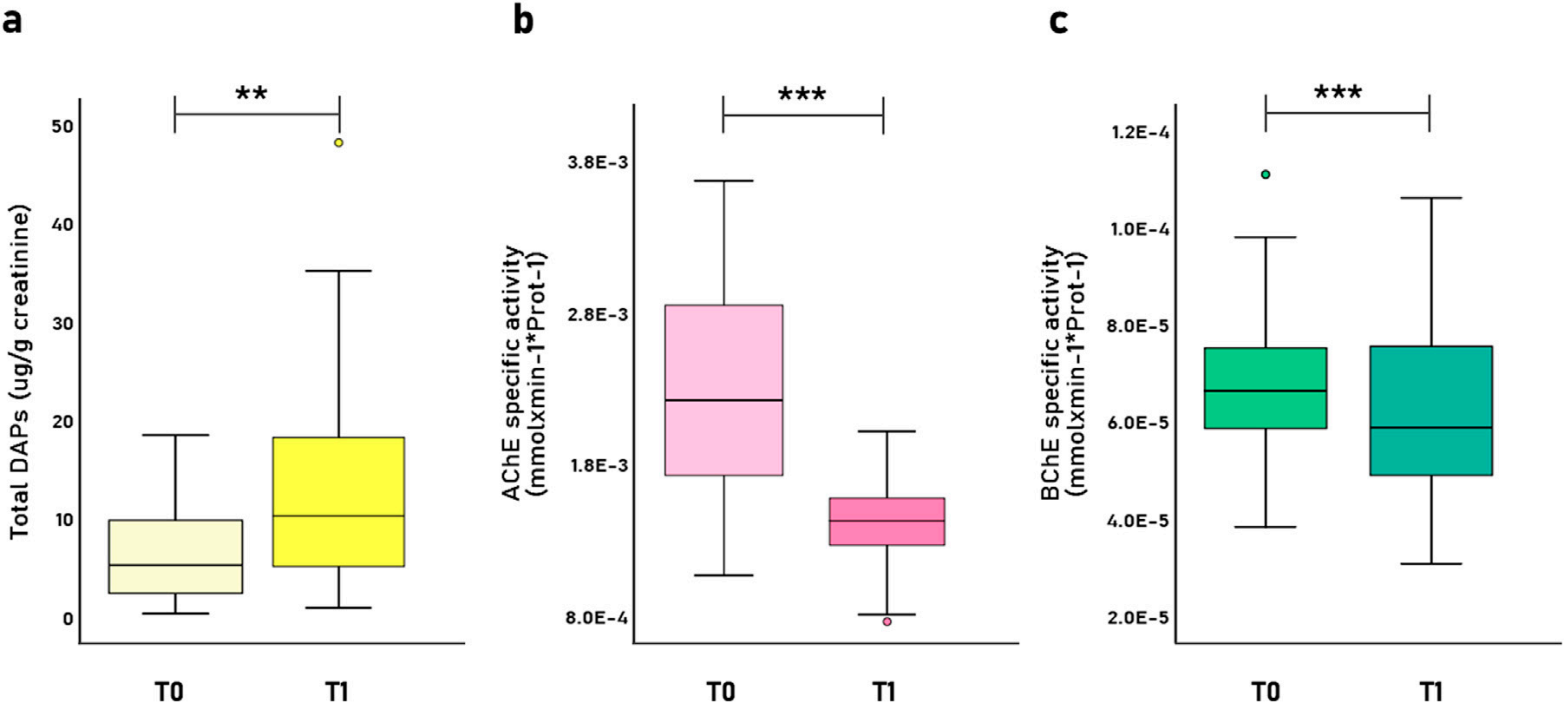
Levels of **(a)** total Dialkyl Phosphate metabolites (ΣDAPs) (μg/g creatinine); **(b)** AChE specific activity (mmol/min^-1^^*^mgProt^-1^), and **(c)** BChE specific activity (mmol/min^-1^^*^mgProt^-1^) during the pre-spray season (T0) and spray season (T1). Boxplot diagrams show the distribution of data (n = 51), including the median, quartiles and outliers. *p*–values were calculated using Wilcoxon signed-rank test; **p<0.01, *** *p*<0.001.

Respect to biomarker analysis and occupational exposure assessment, total urinary DAP levels significantly increased from baseline (T0) to the pesticide spray season (T1) from 6.54 ± 4.66 to 12.39 ± 9.88 μg/g creatinine (Z = −2.89; *p* = 0.004), confirming elevated pesticide exposure during peak application periods (Figure 2a). Of the six DAP metabolites, five were detected (DEP, DETP, DMP, DMTP, and DMDTP), with levels exceeding the 50th percentile of the reference Hispanic population in the United States; additionally, over 70% of participants had urinary DAP metabolite concentrations above reference thresholds, indicating substantial exposure (data not shown).

Figures 2b,c show the decrease in AChE and BChE specific activity during the spray season compared to baseline, with values dropping from 2.26E-3 ± 6.53E-4 to 1.44E-3 ± 2.73E-4 mmol/min^-1^*mgProt^-1^ for AChE activity (Z = −6.13; *p*–value <0.001), and from 6.76E-5 ± 1.50E-5 to 5.86E-5 ± 1.28E-5 mmol/min-1*mgProt-1 for BChE activity (Z = −4.39; *p*–value <0.001). In terms of enzyme inhibition, mean AChE inhibition was 30.99% (SD = 19.74%) exceeding the 30% biological tolerance limit (BTL) established in Chilean regulations (Figures 2b,c), while BChE inhibition averaged 10.74% (SD = 18.11%).

No associations were found between urinary DAP levels and AChE or BChE inhibition. Additionally, bivariate analysis showed that AChE or BChE inhibition were not influenced by sex, age, level of education, length of residence in the agricultural zone, or duration of occupational exposure. Change in total DAP metabolite concentration from T0 to T1 were not modified by the sociodemographic factors (data not shown).

### 3.3 Questionnaire performance

**FIGURE 3:**
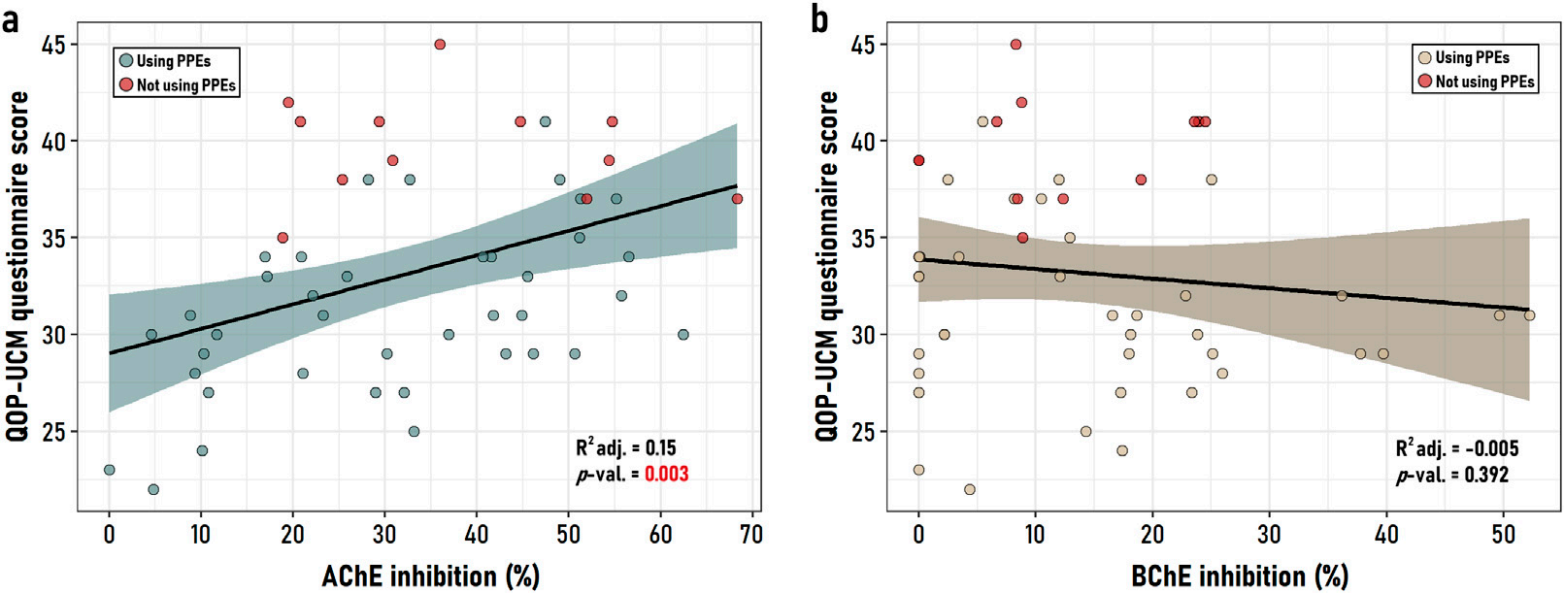
Association between exposure levels assessed with the QOP-UCM and the percentages of **(a)** AChE inhibition and **(b)** BChE inhibition (*n* = 51). Shaded bands represent 95% confidence intervals. Data points are categorized according to personal protective equipment (PPE) usage. Note the difference in the range of the x-axis between panels.

The QOP-UCM questionnaire performance demonstrated good internal consistency across its 37 items, with Cronbach’s alpha = 0.78. The mean questionnaire score was 33.06 points (SD = 5.3), while the median was 33 points (IQR: 25% = 29 points and 75% = 37.5 points). In the association analysis, the QOP-UCM score was not related to DAP concentration in urine (data not shown). Conversely, a positive correlation was found between the QOP-UCM score and AChE inhibition (Pearson r = 0.41; *p* = 0.002) (Figure 3a), but not with BChE inhibition (Pearson r = −0.11; *p* = 0.395) (Figure 3b).

### 3.4 Biomarkers vs. QOP-UCM

The role of PPE use is highlighted in Figure 3, indicating that those workers who did not use PPE were more likely to exhibit higher AChE inhibition and higher QOP- UCM scores. Table 2 shows that both AChE inhibition (β = 0.10; *p*–value = 0.006) and the lack of PPE use (β = 8.11; *p*–value <0.001) significantly contributed to the QOP-UCM scores obtained in the study group.

**TABLE 2:** Variables associated with the estimation of QOP-UCM questionnaire scores based on acetylcholinesterase inhibition, adjusted for covariates.

Variable	Coefficient (β)	Standard error	t	*p*-value
AChE inhibition	0.088	0.03	2.86	**0.0063**
Residence time	−0.06	0.04	−1.59	0.1184
Age	0.07	1.25	1.39	0.1712
Sex (Female)^a^	1.28	1.22	1.05	0.2984
PPE (not use)^a^	8.11	1.26	6.41	**< 0.0001**

^a^ The information in parentheses corresponds to the reference group.

PPE, personal protective equipment; AChE, acetylcholinesterase.

The model was built using the “enter” method for variable selection; all variables in a single step. Bold values indicate statistically significant p-values (p < 0.05).

According to Muñoz-Quezada et al. (2019a), a positive questionnaire result is defined as a median score equal to or greater than 32. The contingency distribution of workers based on these criteria is presented in Table 3, considering groups formed by those with AChE inhibition levels below and above 30% and 20%.

**TABLE 3:** Contingency distributions QOP-UCM scores (cutoff score = 32) and acetylcholinesterase inhibition levels (30% and 20%) including analysis of sensitivity, specificity, and predictive values of the questionnaire for exposure status.

QOP - UCM score distribution	Distribution of AChE by BTL			Sensitivity	Specificity	PPV	NPV	AUC (95%CI)
	Positive ≥30%	Negative <30%	Total	0.64	0.56	0.62	0.58	0.60 (0.45–0.76)
Positive ≥32 score	18	11	29					
Negative <31 score	10	14	24					
Total	28	25	53					
	Positive ≥20%	Negative <20%	Total	0.64	0.71	0.86	0.42	0.68 (0.51–0.84)
Positive ≥32 score	25	4	29					
Negative <31 score	14	10	24					
Total	40	13	53					

AChE, acetylcholinesterase; BTL, biologic tolerance limit; PPV, positive predictive value; NPV, negative predictive value; AUC, area under the curve; CI95%, 95% confidence interval.

### 3.5 Diagnostic performance analysis

**FIGURE 4:**
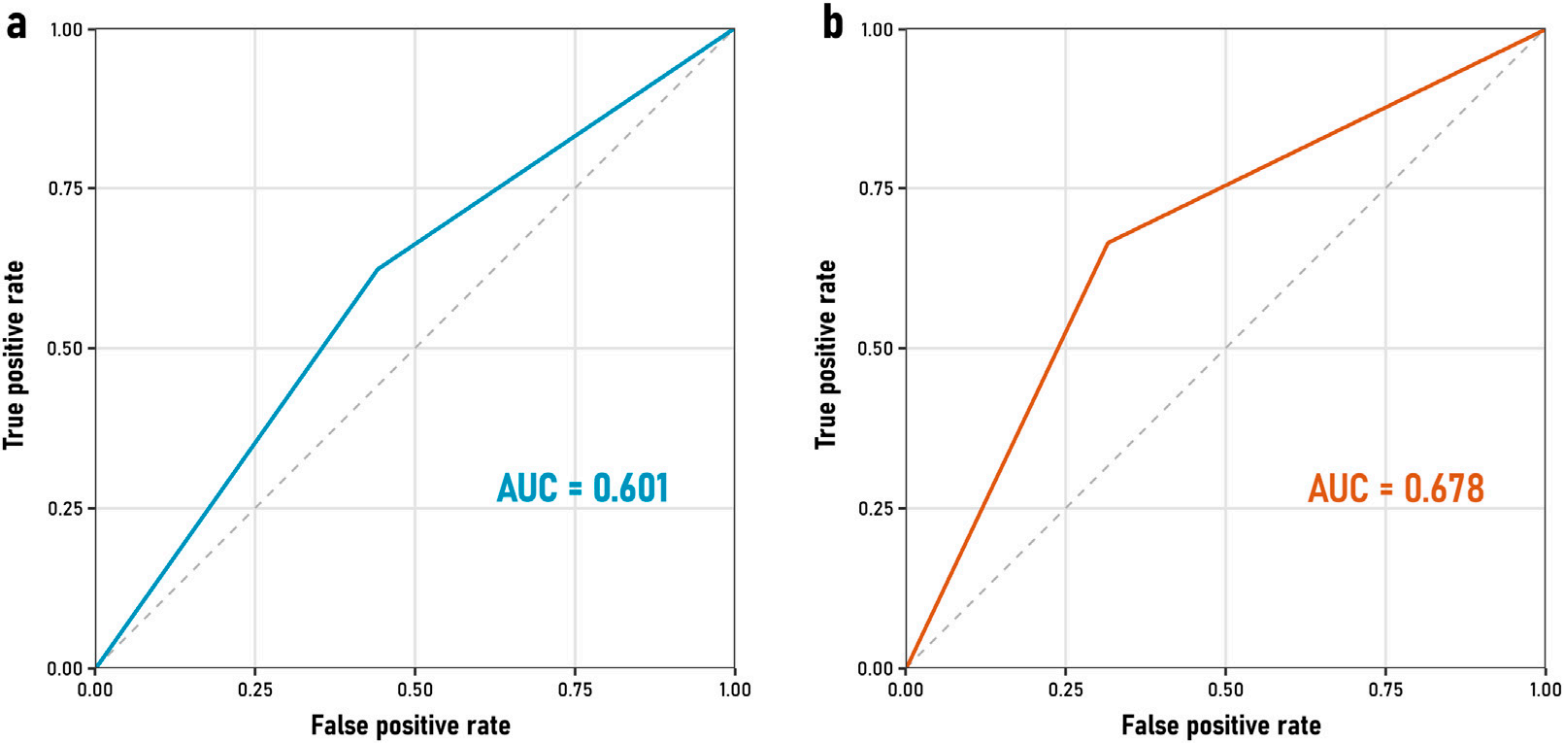
Receiver Operating Characteristic (ROC) curves comparing the performance of the QOP-UCM questionnaire as a classifier against AChE inhibition at **(a)** 30% (AUC = 0.601, blue line) and **(b)** 20% (AUC = 0.678, red line).

When evaluating the diagnostic performance of the questionnaire, sensitivity was 64% for both 30% and 20% AChE inhibition thresholds. However, specificity improved from 56% to 71% when using a 20% inhibition threshold (Table 3). The positive predictive value (PPV) increased from 62% (30% threshold) to 86% (20% threshold), while the negative predictive value (NPV) decreased from 58% to 42%, respectively. These indicators reflect the potential of the questionnaire as a screening tool, demonstrating higher specificity and PPV when considering a 20% AChE inhibition threshold. The agreement between both thresholds is observed by their respective Receiver Operating Characteristic (ROC) curves for the QOP-UCM questionnaire in relation to AChE inhibition at 30% and 20% (Figure 4). These yielded an area under the curve (AUC, a measure of discriminative ability) of 0.6 and 0.68, respectively.

## 4 Discussion

This study evaluates the reliability and predictive validity of the QOP-UCM questionnaire, designed to estimate occupational exposure to OP pesticides, by comparing its results with established biomarkers such as of AChE and BChE inhibition and urinary DAP metabolites among agricultural workers in the Maule region of Chile. Our results provide valuable evidence supporting the utility of the questionnaire as a screening tool for identifying at-risk populations, potentially enhancing monitoring and prevention practices in agricultural settings. Compared to existing tools such as the Argentinian exposure index (Filippi et al., 2021) or hospital-based screening scores (Chaudhary et al., 2019), the QOP-UCM is unique in its integration of chronic and acute exposure domains validated against biological markers. Furthermore, its development was context-specific, targeting smallholder agricultural workers in Chile, a group often excluded from standardized tools designed for industrial agriculture. These features make QOP-UCM both novel and well-adapted for high-risk rural populations in Latin America.

The detection of five out of six urinary DAP metabolites in the study population confirms widespread OP pesticide exposure. Over 70% of participants exhibited metabolite levels exceeding the median values reported for the general Hispanic population in the United States (CDC - Centers of Diseases Control and Prevention, 2018), highlighting the significant exposure burden in this region. This finding aligns with previous research indicating elevated pesticide metabolites levels in children from similar agriculture areas (Muñoz-Quezada et al., 2019b). However, the lack of correlation between urinary DAP levels and cholinesterase inhibition reinforces existing evidence suggesting limitations of DAPs as quantitative predictors of cholinergic effects (Sudakin and Stone, 2011). DAPs are present in various environmental matrices (food, air, water), often at levels exceeding those from OP pesticides, which may lead to overestimation of exposure. Moreover, DAP metabolites do not directly inhibit cholinesterase enzymes, and other cholinesterase-inhibiting pesticides (e.g., carbamates) do not yield DAP metabolites, limiting their specificity in assessing biological impact (Sudakin and Stone, 2011). To our knowledge, there is no evidence from occupational exposure studies supporting a clear relationship between urinary DAP excretion and significant cholinesterase activity inhibition, nor a DAP threshold indicative of cholinergic signs or symptoms in a clinical context. Therefore, while DAPs remain useful as qualitative exposure indicators, they should not be solely relied upon for determining biological impact of OP pesticides (Quandt et al., 2010).

The current regulatory threshold for cholinesterase inhibition warrants reconsideration. Although exceeding the established biological tolerance limit (BTL) necessitates removal from exposure, studies indicate that even inhibition levels above 30% are associated with chronic adverse health effects (Ramírez-Santana et al., 2020a; Ramírez-Santana et al., 2020b). This suggests that lowering the regulatory limit could enhance early detection and prevention of pesticide-related toxicity. In this context, our inclusion of a 20% AChE inhibition threshold serves as an intermediate and evidence-based criterion that improves sensitivity for detecting subclinical effects among exposed individuals. This decision is supported by regulatory agencies like the EPA, which has established a 10% inhibition threshold in erythrocytes as a conservative safety margin to protect against neurotoxic effects of chlorpyrifos (EPA–Environmental Protection Agency. Chlorpyrifos Final Order Denying Objections, 2022), one of the most widely used insecticides in Chile and worldwide. Similarly, the Washington State Department of Labor and Industries initiates workplace investigation when AChE inhibition exceeds 20% and mandates removal from exposure at 30% (Hofmann et al., 2010). Krenz et al. (2015) further support this approach, noting that ChE inhibition levels between 20% and 30% may already indicate relevant subclinical neurotoxic effects and that early interventions based on these thresholds are justified in occupational surveillance programs.

Furthermore, the current practices of measuring AChE and BChE indistinctly may lead to underestimation of exposure and risk, as AChE is more sensitive to chronic exposure while BChE better reflects acute exposure (Assis et al., 2018; Strelitz et al., 2014). A more precise protocol requires individual baseline cholinesterase measurements before and during pesticide application, rather than relying on historical baselines, to account for individual variability and reduce underreporting. Implementing these criteria in occupational health assessments could enhance early risk detection and improve preventive interventions, particularly in vulnerable populations.

The QOP-UCM questionnaire demonstrated a positive association with AChE inhibition, but not with BChE inhibition. This difference likely reflects the questionnaire’s focus on chronic exposure, whereas BChE’s shorter half-life makes it a better indicator of acute effects (Assis et al., 2018). The questionnaire exhibited good internal consistency (Cronbach’s alpha of 0.78), supporting its reliability as an occupational exposure assessment tool. However, while its sensitivity and specificity values indicate potential utility, they also suggest areas for further refinement. The sensitivity of 0.64 at both the 30% and 20% AChE inhibition threshold suggests that some exposed individuals may not be identified, whereas the improved specificity at the 20% threshold (71%) compared to 30% threshold (56%) underscores the need for context-specific thresholds to minimize misclassification (Filippi et al., 2021).

The trade-off between sensitivity and specificity influences predictive values, including positive predictive value (PPV) and negative predictive value (NPV). The PPV reflects the likelihood that an individual identified as exposed by the questionnaire is truly exposed, whereas the NPV represents the probability of being truly unexposed given a negative result. The QOP-UCM’s predictive values further highlight both strength and limitations. The PPV of 86% indicates that the questionnaire effectively identifies workers with significant cholinesterase inhibition. However, the relatively low NPV suggests that additional adjustments are necessary. Although the 32-point threshold for the QOP-UCM score was based on the sample median, future studies may benefit from identifying optimal cut-off points using data-driven metrics such as Youden’s index (Youden, 1950), which maximizes the difference between true positive and false positive rates. This approach could enhance the tool’s diagnostic performance in larger and more heterogeneous populations.

These findings emphasize the importance of modifying scoring algorithms, adding exposure-relevant questions, or incorporating biomarkers into a composite exposure index. Additionally, longitudinal assessments across multiple pesticide application seasons, varying working conditions and safety practices influence the questionnaire’s performance, therefore it could enhance the tool’s predictive capacity, particularly in scenarios where precise identification of exposure is paramount.

We recognize that the sample size may limit the generalizability of the findings to broader agricultural populations, and further validation with larger and more diverse cohorts is needed. Moreover, the study was geographically restricted to the Maule region, with participants primarily working in similar crop types and performing comparable agricultural tasks. Exposure was assessed during a single pesticide spray season, limiting the capacity to evaluate inter-annual variability or other seasonal patterns. These contextual constraints may restrict the external validity of our results. Future studies should aim to replicate and validate the QOP-UCM questionnaire across multiple agroecological zones, crop systems, and labor structures to enhance its applicability.

Additionally, reliance on self-reported exposure data introduces potential recall bias, which could affect the accuracy of exposure assessment.

While AChE inhibition is a widely accepted biomarker of OP exposure, its interpretation has important limitations. Cholinesterase activity is a nonspecific indicator that can be influenced by factors unrelated to pesticide exposure, including nutritional status, hepatic function, genetic polymorphisms, and concomitant medications (Strelitz et al., 2014; Assis et al., 2018). These confounders were not controlled in our analysis, which constitutes a limitation of the study. Given the inter-individual variability in baseline cholinesterase activity, future occupational health monitoring programs should consider establishing personal baseline values before pesticide exposure periods. This practice could enhance early detection of enzyme inhibition and improve the reliability of biological surveillance in high-risk populations.

These recommendations aim to reduce occupational poisoning risk, given that in Chile, approximately 5,000 cases of pesticide poisoning occur annually, excluding underreporting, which is estimated to be 5 to 10 times higher. Around 72% of these cases are work-related (MINSAL - Ministerio de Salud. Gobierno de Chile, 2015). If current policies remain unchanged, the increasing trend in pesticides poisoning cases, along with its high mortality rates and a significant burden on the public health system (Ramírez-Santana et al., 2014), is likely to persist. This scenario could worsen with the projected rise in pesticide use due to climate change.

As a conclusion, these findings are critical for improving occupational health policies and risk assessment strategies in agricultural settings with high pesticide exposure. Implementing validated, cost-effective screening tools, such as the QOP-UCM questionnaire, can facilitate early interventions, reducing pesticide-related morbidity among farm workers. Further research is recommended to expand the questionnaire’s applicability across diverse agricultural populations and exposure conditions.

## Data Availability

The raw data supporting the conclusions of this article will be made available by the authors, without undue reservation.

## References

[B1] Alavanja M. (2009). Introduction: pesticides use and exposure extensive worldwide. Rev. Environ. Health 24, 303–309. 10.1515/reveh.2009.24.4.303 20384038 PMC2946087

[B2] Aroniadou-Anderjaska V. Figueiredo T. de Araujo Furtado M. Pidoplichko V. Braga M. (2023). Mechanisms of organophosphate toxicity and the role of acetylcholinesterase inhibition. Toxics 11, 866. 10.3390/toxics11100866 37888716 PMC10611379

[B3] Assis C. Linhares A. Cabrera M. Oliveira V. Silva K. Marcuschi M. (2018). Erythrocyte acetylcholinesterase as biomarker of pesticide exposure: new and forgotten insights. Environ. Sci. Pollut. Res. Int. 25, 18364–18376. 10.1007/s11356-018-2303-9 29797194

[B4] CDC - Centers of Diseases Control and Prevention (2018). National report on human exposure to environmental chemicals. Atlanta, GA: U.S. Department of Health and Human Services. Available online at: https://www.cdc.gov/exposurereport/data_tables.html?NER_SectionItem=NHANES.

[B5] Chambers J. Chambers H. (1990). Time course of inhibition of acetylcholinesterase and aliesterases following parathion and paraoxon exposures in rats. Toxicol. Appl. Pharmacol. 103, 420–429. 10.1016/0041-008x(90)90315-l 2339415

[B6] Chaudhary R. Bhandari R. Malla G. (2019). Correlation of clinical score and serum acetylcholinesterase level as a predictor of outcome among patients with acute organophosphate poisoning. J. BP Koirala Inst. Health Sci. 3, 89–95. 10.3126/jbpkihs.v2i2.27853

[B7] Coble J. Arbuckle T. Lee W. Alavanja M. Dosemeci M. (2005). The validation of a pesticide exposure algorithm using biological monitoring results. J. Occup. Environ. Hyg. 2, 194–201. 10.1080/15459620590923343 15764542

[B9] Ellman G. Courtney K. Andres V. Feather-Stone R. (1961). A new and rapid colorimetric determination of acetylcholinesterase activity. Biochem. Pharmacol. 7, 88–95. 10.1016/0006-2952(61)90145-9 13726518

[B10] EPA – Environmental Protection Agency. Chlorpyrifos; Final Order Denying Objections (2022). Requests for hearings, and requests for a stay of the August 2021 tolerance final rule. Fed. Regist. Available online at: https://www.govinfo.gov/content/pkg/FR-2022-02-28/pdf/2022-04139.pdf.

[B11] FAO – Food and Agriculture Organization – Chile (2024). Chile en una mirada. Organización de las Naciones Unidas para la Alimentación y la Agricultura. Available online at: https://www.fao.org/chile/fao-en-chile/chileunamirada/es/.

[B12] Filippi I. Lucero P. Bonansea R. Lerda D. Butinof M. Fernandez R. A. (2021). Validation of exposure indexes to pesticides through the analysis of exposure and effect biomarkers in ground pesticide applicators from Argentina. Heliyon 7, e07921. 10.1016/j.heliyon.2021.e07921 34522813 PMC8427256

[B13] Hofmann J. N. Keifer M. C. De Roos A. J. Fenske R. A. Furlong C. E. van Belle G. (2010). Occupational determinants of serum cholinesterase inhibition among organophosphate-exposed agricultural pesticide handlers in Washington state. Occup. Environ. Med. 67 (6), 375–386. 10.1136/oem.2009.046391 19819864 PMC2908529

[B14] Hornung R. Reed L. (1990). Estimation of average concentration in the presence of nondetectable values. Appl. Occup. Environ. Hyg. 5, 46–51. 10.1080/1047322x.1990.10389587

[B15] ISPCH - Instituto de Salud Pública de Chile (2014). Protocolo. examen de salud para aplicaciones de plaguicidas Protocol. Health Exam. pesticide Appl. Available online at: https://www.minsal.cl/sites/default/files/Protocolo_de_Vigilancia_Trabajadores_Expuestos_Plaguicidas.pdf (Accessed February 10, 2018).

[B16] Jayatilaka N. Restrepo P. Davis Z. Vidal M. Calafat A. M. Ospina M. (2019). Quantification of 16 urinary biomarkers of exposure to flame retardants, plasticizers, and organophosphate insecticides for biomonitoring studies. Chemosphere 235, 481–491. 10.1016/j.chemosphere.2019.06.181 31272008 PMC6960943

[B17] Johnstone K. Capra M. Newman B. (2007). Organophosphate pesticide exposure in agricultural workers: human exposure and risk assessment. Barton, A.C.T., Australia: RIRDC.

[B18] Kapka-Skrzypczak L. Cyranka M. Skrzypczak M. Kruszewski M. (2011). Biomonitoring and biomarkers of organophosphate pesticides exposure - state of the art. Ann. Agric. Environ. Med. AAEM 18, 294–303. 22216802

[B19] Krenz J. E. Hofmann J. N. Smith T. R. Cunningham R. N. Fenske R. A. Simpson C. D. (2015). Determinants of butyrylcholinesterase inhibition among agricultural pesticide handlers in Washington state: an update. Ann. Occup. Hyg. 59 (1), 25–40. 10.1093/annhyg/meu072 25261454 PMC4290628

[B20] Lott E. Jones E. (2022). “Cholinergic toxicity,” Treasure island, FL: StatPearls Publishing (StartPearls). Available online at: https://www.ncbi.nlm.nih.gov/books/NBK539783/. 30969605

[B21] MINSAL - Ministerio de Salud. Gobierno de Chile (2015). Reglamento sobre condiciones para la seguridad sanitaria de las personas en la aplicación terrestre de plaguicidas agrícolas. Available online at: https://www.bcn.cl/leychile/navegar?idNorma=1078695.

[B22] MINSAL - Ministerio de Salud. Gobierno de Chile (2023). Boletín Epidemiológico Trimestral. Intoxicaciones Agudas por Plaguicidas. Available online at: https://epi.minsal.cl/wp-content/uploads/2024/03/2024.03.15_INFORME-REVEP-ANO-2023-TOTAL-Dra.CVS-OF.VENT_.DEPTO_.EPIDEMIOLOGIA-MINSAL.pdf.

[B23] Muñoz-Quezada M. Lucero B. Bradman A. Baumert B. Iglesias V. Muñoz M. P. (2019a). Reliability and factorial validity of a questionnaire to assess organophosphate pesticide exposure to agricultural workers in Maule, Chile. Int. J. Environ. Health Res. 29, 45–59. 10.1080/09603123.2018.1508647 30124052

[B24] Muñoz-Quezada M. Lucero B. Bradman A. Steenland K. Zúñiga L. Calafat A. (2019b). An educational intervention on the risk perception of pesticides exposure and organophosphate metabolites urinary concentrations in rural school children in Maule region, Chile. Environ. Res. 176, 108554. 10.1016/j.envres.2019.108554 31288198 PMC7953381

[B25] Ohlander J. Fuhrimann S. Basinas I. Cherrie J. Galea K. Povey A. (2020). Systematic review of methods used to assess exposure to pesticides in occupational epidemiology studies, 1993-2017. Occup. Environ. Med. 77, 357–367. 10.1136/oemed-2019-105880 32098789 PMC7279185

[B26] Oltramare C. Mediouni Z. Shoman Y. Hopf N. Graczyk H. Berthet A. (2023). Determinants of pesticide exposure in occupational studies: a meta-analysis. Toxics 11, 623. 10.3390/toxics11070623 37505588 PMC10386710

[B27] Panis C. Kawassaki A. Crestani A. Pascotto C. Bortoloti D. Vicentini G. (2022). Evidence on human exposure to pesticides and the occurrence of health hazards in the Brazilian population: a systematic review. Front. Public Health 9, 787438. 10.3389/fpubh.2021.787438 35071167 PMC8777228

[B28] Quandt S. Chen H. Grzywacz J. Vallejos Q. Galvan L. Arcury T. (2010). Cholinesterase depression and its association with pesticide exposure across the agricultural season among Latino farmworkers in North Carolina. Environ. Health Perspect. 118, 635–639. 10.1289/ehp.0901492 20085857 PMC2866678

[B29] Ramírez-Santana M. Farías-Gómez C. Zúñiga-Venegas L. Sandoval R. Roeleveld N. Van der Velden K. (2018). Biomonitoring of blood cholinesterases and acylpeptide hydrolase activities in rural inhabitants exposed to pesticides in the Coquimbo region of Chile. PloS one 13 (5), e0196084. 10.1371/journal.pone.0196084 29718943 PMC5931667

[B30] Ramírez-Santana M. Iglesias-Guerrero J. Castillo-Riquelme M. Scheepers P. T. J. (2014). Assessment of health care and economic costs due to episodes of acute pesticide intoxication in workers of rural areas of the Coquimbo region, Chile. Value Health Reg. issues. 5, 35–39. 10.1016/j.vhri.2014.07.006 29702785

[B31] Ramírez-Santana M. Zúñiga L. Corral S. Sandoval R. Scheepers P. Van der Velden K. (2015). Assessing biomarkers and neuropsychological outcomes in rural populations exposed to organophosphate pesticides in Chile-study design and protocol. BMC Public Health 15, 116. 10.1186/s12889-015-1463-5 25881174 PMC4358855

[B32] Ramírez-Santana M. Zúñiga-Venegas L. Corral S. Roeleveld N. Groenewoud H. Van der Velden K. (2020a). Reduced neurobehavioral functioning in agricultural workers and rural inhabitants exposed to pesticides in northern Chile and its association with blood biomarkers inhibition. Environ. Health a Glob. Access Sci. Source 19, 84. 10.1186/s12940-020-00634-6 PMC7374955 32698901

[B33] Ramírez-Santana M. Zúñiga-Venegas L. Corral S. Roeleveld N. Groenewoud H. van der Velden K. (2020b). Association between cholinesterase's inhibition and cognitive impairment: a basis for prevention policies of environmental pollution by organophosphate and carbamate pesticides in Chile. Environ. Res. 186, 109539. 10.1016/j.envres.2020.109539 32361078

[B34] SAG - Servicio Agrícola y Ganadero (2023). Reporte general Ventas de Plaguicidas de uso agrícola en Chile. División Protección Agrícola y Forestal. Sección Inocuidad. Ministerio de Agricultura. Santiago, Chile: Gobierno de Chile. Available online at: https://app.powerbi.com/view?r=eyJrIjoiOGMxOWM5MDctNWVlMC00MGQyLTkxMDEtYjY3YTQzMGUxMjkyIiwidCI6Ijc3ZWNkYTc1LTU4NjQtNDIyYS1hNTM1LTZlYTY3MTU0MDI5YyIsImMiOjR9.

[B35] Shekhar C. Khosya R. Thakur K. Mahajan D. Kumar R. Kumar S. (2024). A systematic review of pesticide exposure, associated risks, and long-term human health impacts. Toxicol. Rep. 13, 101840. 10.1016/j.toxrep.2024.101840 39717852 PMC11664077

[B36] Smith P. Krohn R. Hermanson G. Mallia A. Gartner F. Provenzano M. (1985). Measurement of protein using bicinchoninic acid. Anal. Biochem. 150, 76–85. 10.1016/0003-2697(85)90442-7 3843705

[B37] Sontakke T. Kalantri S. (2023). Predictors of mortality in hospitalized patients with pesticide poisoning. Cureus 15, e27074. 10.7759/cureus.41284 PMC10393197 37533608

[B38] Strelitz J. Engel L. Keifer M. (2014). Blood acetylcholinesterase and butyrylcholinesterase as biomarkers of cholinesterase depression among pesticide handlers. Occup. Environ. Med. 71, 842–847. 10.1136/oemed-2014-102315 25189163 PMC4224972

[B39] Sudakin D. Stone D. (2011). Dialkyl phosphates as biomarkers of organophosphates: the current divide between epidemiology and clinical toxicology. Clin. Toxicol. Phila. Pa. 49, 771–781. 10.3109/15563650.2011.624101 22077242

[B40] Ueyama J. Saito I. Kondo T. Taki T. Kimata A. Saito S. (2012). Urinary concentrations of organophosphorus insecticide metabolites in Japanese workers. Chemosphere 87, 1403–1409. 10.1016/j.chemosphere.2012.02.048 22455950

[B41] WHO - World Health Organization (2019). WHO recommended classification of pesticides by hazard and guidelines to classification. Geneva, Switzerland: World Health Organization. Available online at: https://iris.who.int/bitstream/handle/10665/332193/9789240005662-eng.pdf.

[B42] Youden W. J. (1950). Index for rating diagnostic tests. Cancer 3 (1), 32–35. 10.1002/1097-0142(1950)3:1<32::aid-cncr2820030106>3.0.co;2-3 15405679

[B43] Zúñiga-Venegas L. Hyland C. Muñoz-Quezada M. Quirós-Alcalá L. Butinof M. Buralli R. (2022). Health effects of pesticide exposure in Latin American and the Caribbean populations: a scoping review. Environ. Health Perspect. 130, 96002. 10.1289/EHP9934 36173136 PMC9521041

