# Supplementary material for: Validation of a novel questionnaire for assessing occupational exposure to organophosphate pesticides in Chilean agricultural workers
Source: Front Toxicol. 2025 Aug 18;7:1588408. doi: 10.3389/ftox.2025.1588408 (PMC12399619; doi:10.3389/ftox.2025.1588408)
Supplement: Supplementary file 1 [file Table1.docx]

| **Supplementary Table 1:** Questionnaire for assessing occupational exposure to OP pesticides in farmworkers from Maule region in Chile (QOP-UCM). | | | |
| --- | --- | --- | --- |
| **Question** | **Answer score** | **Frequency** | **Percentage** |
| Are you usually working applying pesticides? | No (0) | 6 | 10.5 |
|  | Yes (1) | 51 | 89.5 |
| When was your last pesticide application? | Not applicable (0) | 0 | 0 |
|  | 2 years or more (1) | 3 | 5.3 |
|  | Less than 2 years (2) | 54 | 94.7 |
| Do you work as a seasonal or permanent pesticide applicator? | Not applicable (0) | 0 | 0 |
|  | Seasonally (1) | 43 | 75.4 |
|  | Permanent (2) | 14 | 24.6 |
| Do you have a pesticide applicator license? | Not applicable (0) | 0 | 0 |
|  | Yes (1) | 15 | 26.3 |
|  | No (2) | 42 | 73.7 |
| Do you eat, drink or smoke during the application? | Not applicable (0) | 0 | 0 |
|  | No (0) | 55 | 96.5 |
|  | Yes/occasionally (1) | 2 | 3.5 |
| Do you change your clothes after the application? | Not applicable (0) | 0 | 0 |
|  | Yes (1) | 42 | 73.7 |
|  | No/occasionally (2) | 15 | 26.3 |
| The place where pesticides are mixed is | Not applicable (0) | 2 | 3.5 |
|  | Open (1) | 49 | 86.0 |
|  | Closed (2) | 6 | 10.5 |
| Do you use manual pump backpack to apply OPs? | No (0) | 18 | 31.6 |
|  | Yes (1) | 39 | 68.4 |
| What type of OP do you recall applying? | Not applicable (0) | 0 | 0 |
|  | Only one OP (1) | 24 | 42.1 |
|  | More than one OP (2) | 33 | 57.9 |
| Do you wear personal protective equipment when mixing? | Not applicable (0) | 2 | 3.5 |
|  | Yes (1) | 39 | 68.4 |
|  | No (2) | 16 | 29.1 |
| Do you know the health risks you are exposed to when applying or mixing pesticides? | Not applicable (0) | 0 | 0 |
|  | Yes (1) | 48 | 84.2 |
|  | No (2) | 9 | 15.8 |
| How many years have you applied pesticides? | Not applicable (0) | 0 | 0 |
|  | 10 years or less (1) | 7 | 12.3 |
|  | More than 10 years (2) | 50 | 87.7 |
| How long does it take between the end of the application and taking a shower or bath? | Not applicable (0) | 0 | 0 |
|  | Less than 15 min (1) | 25 | 43.9 |
|  | 15 min or more (2) | 32 | 56.1 |
| During or after pesticide application, do you wash your hands before smoking, eating, or drinking? | Not applicable (0) | 0 | 0 |
|  | Yes (1) | 57 | 100 |
|  | No/occasionally (2) | 0 | 0 |
| If you change your clothes after work, where do you do it? | Not applicable (0) | 3 | 5.3 |
|  | At work (1) | 19 | 33.3 |
|  | At home (2) | 35 | 61.4 |
| Are you trained in the health risks of pesticides? | Not applicable (0) | 0 | 0 |
|  | Yes (1) | 35 | 61.4 |
|  | No (2) | 22 | 38.6 |
| Where do you wash the machinery? | Does not wash/Not applicable (0) | 2 | 3.5 |
|  | In a dedicated place for washing (1) | 33 | 57.9 |
|  | Yard, orchard, pasture, or field (2) | 22 | 38.6 |
|  | Inside the house (3) | 0 | 0 |
| Besides applying OPs, do you mix and prepare them? | No or not applicable (0) | 4 | 7.0 |
|  | Yes (1) | 53 | 93.0 |
| Where do you store the pesticides? | Not applicable (0) | 0 | 0 |
|  | Warehouse at home or work (1) | 55 | 96.5 |
|  | In the house ‘s yard (2) | 1 | 1.8 |
|  | Inside the house (3) | 1 | 1.8 |
| Do you use a motorized pump backpack to apply OPs? | No (0) | 31 | 54.4 |
|  | Yes (1) | 26 | 45.6 |
| How long have you worked as an agricultural worker? | 10 years or less (0) | 7 | 12.3 |
|  | More than 10 years (1) | 50 | 87.7 |
| Do you use a tractor operated nebulizer or pump to apply OPs? | No (0) | 29 | 50.9 |
|  | Yes (1) | 28 | 49.1 |
| Use of PPE in hands | Yes (0) | 41 | 71.9 |
|  | No (1) | 16 | 28.1 |
| Use of respiratory PPE | Yes (0) | 38 | 66.7 |
|  | No (1) | 19 | 33.3 |
| Use of protective eyewear | Yes (0) | 31 | 54.4 |
|  | No (1) | 26 | 45.6 |
| Use of PPE in the body | Yes (0) | 31 | 54.4 |
|  | No (1) | 26 | 45.6 |
| Use of PPE in the head | Yes (0) | 44 | 77.2 |
|  | No (1) | 13 | 22.8 |
| Frequency of use of PPE | Always (0) | 34 | 59.6 |
|  | Never or occasionally (1) | 23 | 40.4 |
| Use of PPE in the feet | Yes (0) | 37 | 64.9 |
|  | No (1) | 20 | 35.1 |
| In our workplace we have washbasin | Yes (0) | 50 | 87.7 |
|  | No (1) | 7 | 12.3 |
| In our workplace, we have toilet | Yes (0) | 44 | 77.2 |
|  | No (1) | 13 | 22.8 |
| In our workplace we have showers | Yes (0) | 42 | 73.7 |
|  | No (1) | 15 | 26.3 |
| In our workplace we have drinking water | Yes (0) | 45 | 78.9 |
|  | No (1) | 12 | 21.1 |
| In our workplace, we have hot water | Yes (0) | 28 | 49.1 |
|  | No (1) | 29 | 50.9 |
| Use organophosphate pesticides at home | No (0) | 10 | 17.5 |
|  | Yes (1) | 47 | 82.5 |
| Approximate distance from farm to house (in m) | More than 500 m (0) | 2 | 3.5 |
|  | 500 m or less (1) | 55 | 96.5 |
| Do you have a greenhouse, orchard, or field at home? | No (0) | 16 | 28.1 |
|  | Yes (1) | 41 | 71.9 |
|  |  |  |  |
